# Gershgorin circle theorem-based feature extraction for biomedical signal analysis

**DOI:** 10.3389/fninf.2024.1395916

**Published:** 2024-05-16

**Authors:** Sahaj A. Patel, Rachel June Smith, Abidin Yildirim

**Affiliations:** Department of Electrical and Computer Engineering, University of Alabama at Birmingham, Birmingham, AL, United States

**Keywords:** Gershgorin circle theorem, visibility graph, weighted Laplacian matrix, biomedical signals, deep learning, feature extraction

## Abstract

Recently, graph theory has become a promising tool for biomedical signal analysis, wherein the signals are transformed into a graph network and represented as either adjacency or Laplacian matrices. However, as the size of the time series increases, the dimensions of transformed matrices also expand, leading to a significant rise in computational demand for analysis. Therefore, there is a critical need for efficient feature extraction methods demanding low computational time. This paper introduces a new feature extraction technique based on the Gershgorin Circle theorem applied to biomedical signals, termed Gershgorin Circle Feature Extraction (GCFE). The study makes use of two publicly available datasets: one including synthetic neural recordings, and the other consisting of EEG seizure data. In addition, the efficacy of GCFE is compared with two distinct visibility graphs and tested against seven other feature extraction methods. In the GCFE method, the features are extracted from a special modified weighted Laplacian matrix from the visibility graphs. This method was applied to classify three different types of neural spikes from one dataset, and to distinguish between seizure and non-seizure events in another. The application of GCFE resulted in superior performance when compared to seven other algorithms, achieving a positive average accuracy difference of 2.67% across all experimental datasets. This indicates that GCFE consistently outperformed the other methods in terms of accuracy. Furthermore, the GCFE method was more computationally-efficient than the other feature extraction techniques. The GCFE method can also be employed in real-time biomedical signal classification where the visibility graphs are utilized such as EKG signal classification.

## 1 Introduction

In recent years, there has been a substantial increase in the adoption of non-invasive devices for measuring brain activity, such as electroencephalography (EEG) (Minguillon et al., 2017; He et al., 2023). The non-invasiveness and high temporal resolution make it a convenient and essential tool for research and clinical diagnosis of neurological diseases (Perez-Valero et al., 2021). EEG is measured by placing electrodes on the scalp and it provides indispensable insights into the synchronous activity of populations of cortical neurons (David et al., 2002). EEG signals can be used to understand the underlying neural dynamics of cognitive, motor, and pathological phenomena (Rodriguez-Bermudez and Garcia-Laencina, 2015). For example, EEG signals are used in a wide variety of applications such as neuromarketing (Costa-Feito et al., 2023), investigation of sleep architecture (Gu et al., 2023), detection of neurodegenerative conditions such as Alzheimer’s disease (Modir et al., 2023), neurofeedback therapy (Torres et al., 2023), and epileptic seizure detection (Maher et al., 2023). Over time, various linear and non-linear methods have been developed for extracting distinct features from recorded time series signals. Linear methods of feature extraction encompass families of time-frequency domains such as Fourier transformation, Wavelet transformation, and Empirical Mode Decomposition (Körner, 1988; Percival and Walden, 2000). On the other hand, the non-linear methods involve computations of Lyapunov exponents and recurrence networks (Kantz and Schreiber, 2003; Campanharo et al., 2008). As the EEG time series signals are inherently non-stationary and noisy in nature, robust time-series analysis techniques are necessary to capture meaningful patterns and features in the signal.

In recent years, graph theory approaches have gained popularity as an alternative to traditional time-frequency domain methods for analyzing brain signals (Stam and Van Straate, 2012). The graph networks can reveal non-linear characteristics of non-stationary and chaotic signals. In standard graph theory, the graph consists of sets of nodes and edges where the nodes represent the samples or data points of a time series, and the edges represent the connections or distances between two data points. In 2006, Zhang and Small (2006) introduced the representation of time series data into complex graph networks, revealing chaotic or fractal properties of the time series. In 2008, Lacasa et al. (2008) presented the first natural visibility graph (NVG) that converted time series into a graph network. Unlike standard graphs, which are typically constructed based on predefined relationships between data points, visibility graphs convert each data point in a time series into a node and then connect nodes with an edge if they can ‘see’ each other, usually determined by a line of sight criterion over the time series data. The original NVG, as presented by Lucas et al., had unweighted edges, meaning it did not consider the varying scales or magnitudes of the time series data—this resulted in treating the data univariately. In contrast, standard graphs might not inherently represent temporal or sequential data and are often not designed to handle the dynamic scaling that visibility graphs can accommodate. In 2010, Ahmadlou et al. (2010) implemented the first visibility graph on EEG signals for detecting Alzheimer’s disease.

Beyond the NVG, several groups have developed different variants of visibility graphs, such as Horizontal Visibility Graph (HVG) (Luque et al., 2009), Weighted Visibility Graph (WVG) (Supriya et al., 2016), Limited Penetrable Horizontal Visibility Graph (LPHVG) (Gao et al., 2016), and Weighted Dual Perspective Visibility Graph (WDPVG) (Zheng et al., 2021). Each of these methods construct distinct graph topologies based on the provided time series data. To decode and interpret the tropological characteristics of these graphs, they are transformed into a matrix form such as the Adjacency matrix or Laplacian matrix. Later, feature extraction and reduction techniques are applied on these matrices. For instance, Zhang et al. (2022) used the weighted adjacency matrix as a feature representation for classifying different sleep stages using calcium imaging data. In contrast, Mohammadpoory et al. (2023) experimented with various methods to extract features from adjacency matrices such as Graph Index Complexity (GIC), Characteristic Path Length (CPL), Global Efficiency (GE) (Latora and Marchiori, 2001), Local Efficiency (LE) (Latora and Marchiori, 2001), Clustering Coefficients (CC) (Saramäki et al., 2007), and Assortative Coefficient (AC) (Artameeyanant et al., 2017). Supriya et al. (2016) took a different approach and calculated two network properties: modularity (Blondel, 2008) and an average weighted degree (Antoniou and Tsompa, 2008) from the graph. Likewise, Hao et al. (2016) classified EEG seizures by measuring the graph’s “*Average Path Length*” and CC.

Although incorporating techniques that extract multiple features simultaneously characterizes the resulting graph more robustly, it also requires more computational time to perform feature extraction. In addition, as the number of samples rises, computational time also proportionally increases. Therefore, in real-time application of EEG signals processing, we must have low computational cost for preprocessing and feature extraction methodologies that do not compromise accuracy. Driven by this need, this study presents a new feature extraction method with low computational cost for time series in biomedical signal processing. This study utilizes the Gershgorin Circle (GC) theorem (Gershgorin, 1931) as a technique for primary feature extraction.

In 1931, mathematician S. A. Gershgorin introduced the Gershgorin Circle (GC) theorem, a pivotal method for estimating eigenvalue inclusions for a square matrix. The GC theorem offers a straightforward yet powerful technique to approximate the location of eigenvalues by defining circles in the complex plane, centered at the matrix’s diagonal entries with radii determined by the sum of the absolute values of the off-diagonal entries in each row. This approach not only simplifies the understanding of a matrix’s spectral properties but also requires fewer computational operations compared to other eigenvalue estimation methods (Varga, 2010). As a result, the GC theorem has found extensive applications across various fields, such as stability analysis of nonlinear systems (Ortega Bejarano et al., 2018), power grids (Xie et al., 2022), and graph sampling (Wang et al., 2020), demonstrating its versatility and effectiveness. Furthermore, subsequent advancements have refined the theorem, enhancing the precision of the eigenvalue inclusions and bringing them closer to the actual eigenvalues of a matrix. This evolution underscores the theorem’s significant impact on the mathematical and engineering disciplines, offering a reliable and efficient tool for analyzing and interpreting the eigenvalues of square matrices.

**Figure 1:**
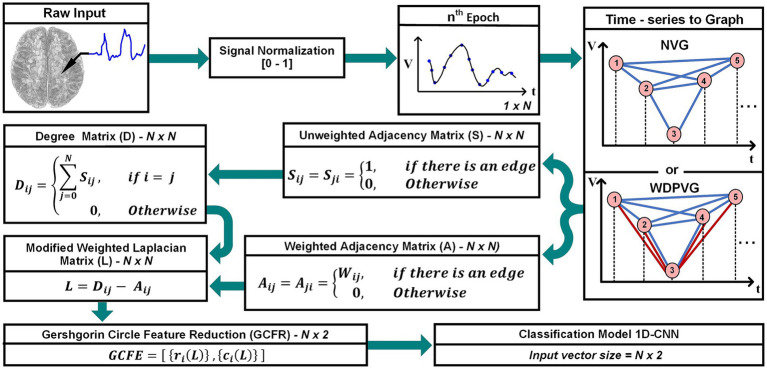
Overall representation of the GCFE framework: from raw signal recording to feature classification.

This study introduces a new, low computational feature extraction approach for time series in biomedical signal processing. In this approach, the GC theorem is used to extract features from a modified Weighted Laplacian (mWL) matrix. Figure 1 shows a block diagram of the GCFE approach. The outline of this paper is as follows: Section 2 explains the proposed approach, which is divided into four subsections: – signal pre-processing, mWL matrix formation, and GCFE and classification model. Section 3 presents a detailed overview of datasets utilized in this study. The GCFE method results, and discussion are described in Section 4. Finally, Section 5 articulates the conclusion of the proposed approach.

## 2 Methodology

The proposed approach can be distilled into four fundamental steps: Preprocessing, forming the mWL matrix, GCFE, and feature classification.

### 2.1 Preprocessing

**Figure 2:**
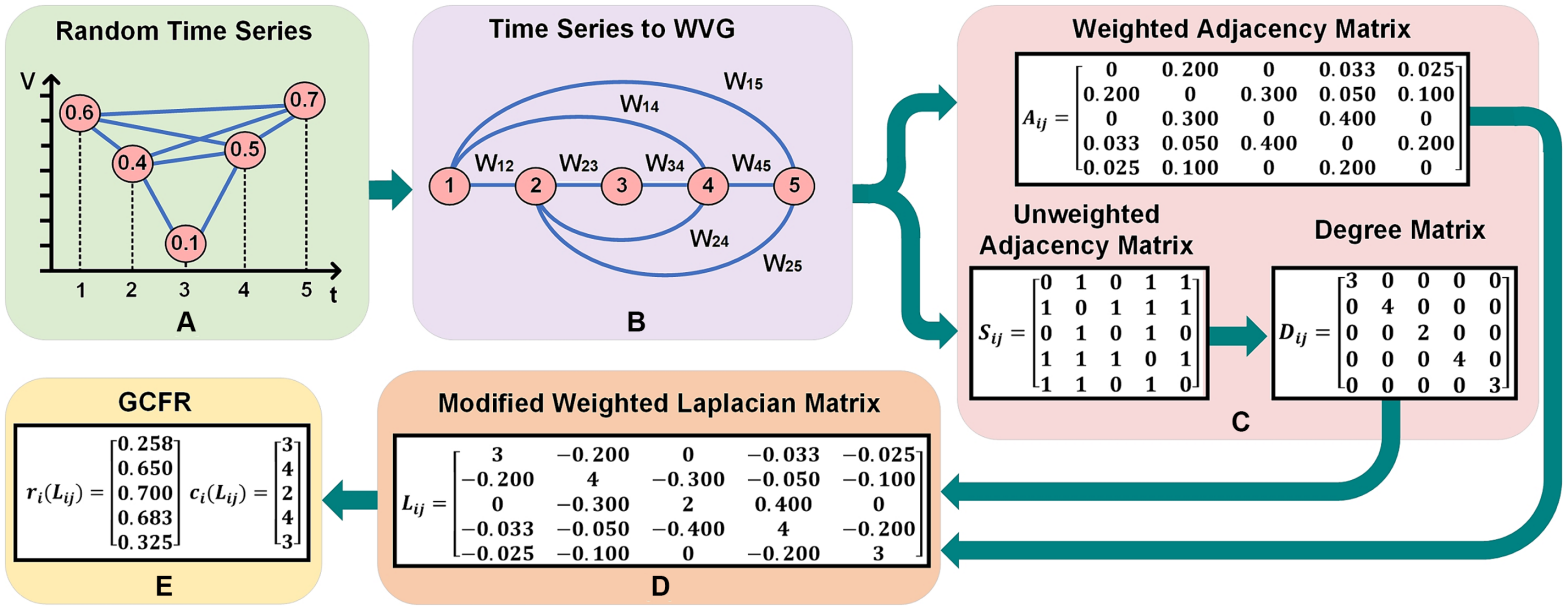
Practical implementation of GCFE on random time series with 5 sample and WVG. **(A)** Shows a random time series with values over discrete time points. **(B)** Depicts the corresponding Weighted Visibility Graph (WVG) representation with weighted edges $W_{ij}$ . **(C)** Illustrates the transition from a weighted adjacency matrix $\;A_{ij}$ , to an unweighted adjacency matrix $\;S_{ij}$ , and a degree matrix $\;D_{ij}$ . **(D)** Presents a modified weighted Laplacian matrix $\;L_{ij}$ derived from the graph. **(E)** Displays the two extracted feature vector by GC Theorem.

In the preprocessing stage, each dataset undergoes into normalization, where the data are scaled between 0 and 1, referenced to raw recording minimum and maximum values. After signal normalization, the whole time series is segmented with N -number of samples with vector size of (1×N) . Each user defined segmented part is called an epoch. In this study, we chose an epoch size of (1×56) for Dataset 1 and (1×1024) for Dataset 2. An example in Figure 2 shows the complete implementation of GCFE for a random time series with five samples and WVG as a graph transformation method. The random time series (*Q*), in Figure 2A represents the normalized values which range between 0 and 1, i.e., *Q* = [0.6, 0.4, 0.1, 0.5, 0.7].

### 2.2 Signal to visibility graph

The next stage is the formation of each epoch into a graph to expose the underlying nonlinear properties of the time series. Two different graph formation methods are utilized to evaluate the performance of the proposed approach across various graph types. Alternative visibility graph transformation techniques beyond WVG and WDPVG can also be integrated into this approach. Any visibility graph consists of a number of nodes and edges, where the nodes represent the data points of the time series, and the edges represent the distance between any two linked nodes. In WVG, only two nodes connect with a weighted edge (denoted as $W_{ij}$ ) if “visibility” between them satisfies the Equation (1).


(1) $$Q\left(t_{z}\right)<Q\left(t_{y}\right)+\left(Q\left(t_{x}\right)-Q\left(t_{y}\right)\right)*\frac{t_{y}-t_{z}}{t_{y}-t_{x}},$$

where, $Q\left(t_{x}\right)$ , $Q\left(t_{y}\right)$ , and $Q\left(t_{z}\right)$ represents the datapoints of a time series with its timestamps $t_{x}$ , $t_{y}$ , and $t_{z}$ respectively. If Equation (1) is satisfied, then the timestamp $t_{z}$ lies in between $t_{x}$ and $t_{y}$ i.e., x<z<y . Then, the weight for each edge is calculated based on Equation (2) (Zheng et al., 2021).


(2) $$Weight\;\left(W_{ij}\right)=\left|\frac{Q\left(i\right)-Q\left(j\right)}{t\left(i\right)-t\left(j\right)}\right|+10^{-8},$$

where, Q(i) and Q(j) are two nodes, t(i) and t(j) are time events of nodes *i* and *j*. Figure 2B shows the conversion of random time series into WVG with weighted node connections $W_{ij}$ . In this example, since node-4 and node-5 are visible for node-1, there are weighted links between node-1 and node-4 (with $W_{14}$ ) and node-1 and node-5 (with $W_{15}$ ). In contrast, the link between node-3 and node-5 is not connected because node-5 is not visible from node-3.

Similar to WVG, the WDPVG is generated by combining two distinct visibility graphs: WVG and Weighted Reflective Perspective Visibility Graph (WRPVG). To form the WDPVG, we first implement the WVG based on Equation (2). Subsequently, the time-series signal is inversed (reflected), after which the nodes are connected again by Equation (2). An illustrative representation of WDPVG can be observed in Figure 1.

### 2.3 Modified weighted Laplacian matrix

To operate with graph networks, it is often necessary to represent these graphs in matrix form. Popular representations of graph networks are the Weighted Adjacency (WA) matrix, the (Unweighted Adjacency) UA matrix, or the Laplacian matrix. This approach introduces a unique modified Weighted Laplacian (mWL), which is a strictly diagonally dominant matrix, and consequently, it inherits Positive Semi-definite (PSD) properties. To generate the mWL matrix, first, the WA (represented by $\;A_{ij}$ in Figure 2C) and UA (represented by $\;S_{ij}\;$ in Figure 2C) matrices are constructed from each WVG or WDPVG. The size of each WA and UA matrix depends upon the number of nodes, which is equivalent to the number of data points in each epoch (N). For instance, in Figure 2B, the WVG has five nodes, as there are five samples in the Q-time series. Note that the WA and UA are square matrices with the size of (N×N) . Both WA and UA matrices are generated according to Equations (3, 4), respectively.


(3) $$A_{ij}=A_{ji}=\{\begin{array}{c} W_{ij},\;\;ifthereis\;an\;edge\\ 0,\;\;otherwise \end{array}$$

(4) $$S_{ij}=S_{ji}=\{\begin{array}{c} 1,\;\;ifthereisanedge\\ 0,\;\;otherwise \end{array}$$

In the WA matrix, the elements of $A_{ij}$ are set to edge weights $W_{ij}$ , if there is an edge between node *i* and *j*; otherwise, the elements are set to 0. Likewise, for the UA matrix, the elements of $S_{ij}$ are assigned a value of “1” if there is an edge between node *i* and *j*, and “0” otherwise. Afterward, the Degree matrix $D_{ij}$ is calculated from the UA matrix as per Equation (5). In the Degree matrix, the diagonal values represent the row summation of all values of the UA matrix. In Figure 2C, an example is presented for $D_{ij}$ , $\;A_{ij},$ and $S_{ij}$ matrices (5 × 5).

The mWL matrix $L_{ij}$ is computed by taking the difference between the Degree matrix ( $D_{ij})\;$ and WA matrix $\left(A_{ij}\right)$ according to Equation (6). Note that the size of the mWL matrix is similar to the WA matrix, i.e., ( N×N ). For example, the mWL matrix $\left(L_{ij}\right)$ shown in Figure 2D is strictly diagonally dominant because its diagonal elements exceed the absolute sum of the corresponding row values.


(5) $$D_{ij}=\{\begin{array}{c} \overset{N}{\underset{j=0}{\sum }}S_{ij},\;\;if\;i=j\\ 0,\;\;Otherwise \end{array}$$

(6) $$L_{ij}=D_{ij}-A_{ij}$$

### 2.4 Gershgorin circle feature extraction

After computing the mWL matrix, the GC theorem is applied to extract features from each $L_{ij}\;$ matrix. The GC theorem states that all the eigenvalues of the ( N×N ) square matrix lies inside the Gershgorin union disks (i.e., Gershgorin circles). The formation of each Gershgorin disk relies on a center point and its radius. The radius of each disk $r_{i}\left(L\right)\;\;$ is computed by taking the absolute row summation of off-diagonal values of ( N×N ) matrix as described in Equation (7). The center of each disk $c_{i}\left(L_{ij}\right)$ is the diagonal value of each row as per Equation (8),


(7) $$r_{i}\left(L_{ij}\right)=\underset{j\in N\backslash \left\{i\right\}}{\sum }|L_{i,j}|\;\left(i\in N\right),$$

(8) $$c_{i}\left(L_{ij}\right)=L_{i,j},\;\;where\;\;i=j\;\;\left(i\in N\right),$$

where the sets of $r_{i}\left(L_{ij}\right)$ and $c_{i}\left(L_{ij}\right)$ represents the GCFE. The final output of each $L_{ij}\;$ matrix is oriented in a vector form, such that all sets of GC are radii, followed by GC centers. This leads to the transformation of $L_{ij}\;$ matrix features, which is in ( N×N ) form, into ({ $r_{i}\left(L_{ij}\right)\}$ x $\left\{c_{i}\left(L_{ij}\right)\right\}$ ) or (2 ×N ) vector form. For instance, the *Q* time series in Figure 2E delivers 10 total GC extracted features, which were $r_{i}\left(L_{ij}\right)=\left[0.258,\;\;0.650,0.700,0.683,0.325\right]$ and $c_{i}\left(L_{ij}\right)=\left[3,4,2,4,3\right]$ respectively.

### 2.5 Feature classification

With the ongoing advancements in machine learning and deep learning, numerous state-of-the-art algorithms have been developed for classifying features. Popular algorithms include but are not limited to, Support Vector Machines (SVM), Decision Trees, and Convolutional Neural Networks (CNN). For this study, the 1D-CNN model was selected to classify the extracted features. However, the proposed method is not limited to using CNN models for feature classification. Other classification methods, such as SVM, Decision Trees, and Artificial Neural Networks (ANN), can also be employed; however, these methods typically require more computational time as the size of the input time-series or the vector size of the extracted features increases.

Table 1 summarizes the architecture of the 1D-CNN model. Dataset – 1 and Dataset – 2 employ the same architecture model, distinguished only by the number of convolution and pooling layers. To classify the features, the GCFE sets are supplied into the 1D-CNN model and then trained according to the target properties. The size of the initial Input Layer depends upon the number of GCFE sets, i.e., batch size ({ $r_{i}\left(L\right)\}$ x $\left\{c_{i}\left(L\right)\right\}$ ), and channels. The Input Layer is followed by connecting sets of the Convolution Layer + Pooling Layer. For Dataset – 1 and Dataset – 2, two and six sets of Convolution Layer and Pooling Layer are used, respectively. Each Convolution Layer utilized 32 filters, with a kernel size of 3, and ReLU was used as an activation function. In the Pooling Layers, the Max Pooling technique was used.

**Table 1:** 1D-CNN architecture and each layer’s configurations.

No. of layers	Layer name	Layer configuration
1	Input layer	(Batch size, rows, channels)
2 or 6	Convolution layer	Conv1D – Kernel = 3, Padding = “same,” Activation Function = “ReLU,” No. Filters = 32
	Pooling layer	MaxPooling1D
1	Flatten layer	(Batch, Flatten last Pooling Layer input size)
1	Dense Layer 1	(Batch Size, 100), Activation Function = “ReLU”
1	Dropout Layer	0.1
1	Dense Layer 2	(Batch Size, 100), Activation Function = “ReLU”
1	Output Layer	(Batch Size, Classes), Activation Function = “SoftMax”

After the final Pooling Layer, a Flatten Layer was connected to transform the feature map (filters) into a 1D vector. Next, the Fully Connected Network (FCN) was built by joining two Dense Layers and one Dropout Layer between the two Dense Layers. Both Dense Layers consisted of 100 artificial neurons and a ReLU activation function. To prevent overfitting, a 10% dropout value was chosen. The final layer of the FCN connects to the Output Layer, the size of which varies based on the dataset classes. For the Output Layer, the SoftMax activation function was used. Note that the 1D-CNN model uses “SparseCategoricalCrossentropy” as its loss function and “Adam” as the optimizer. The detailed mathematical exploration of CNN can be found in Krizhevsky et al. (2012).

## 3 Datasets

Two publicly available datasets are utilized to evaluate the performance of the proposed methodology. The selection of these datasets is strategic, aimed at validating the proposed method on distinct types of signals: simulator-generated action potentials for intracellular recordings and non-invasive EEG recordings, which typically feature a larger number of samples in each epoch. Table 2 details the total number of epochs for both datasets, facilitating a comprehensive assessment of the method’s applicability to different biomedical signals.

**Table 2:** Epoch distribution across datasets and signal-to-noise ratios (snrs) for different classes and sets.

Dataset	No. of epochs			
Dataset – 1	N-class_1	N-class_2	N-class_3	N- class_4
SNR0.5	6,423	6,595	6,597	6,000
SNR1.25	6,394	6,587	6,597	6,000
SNR2.0	5,553	6,633	6,597	6,000
Dataset – 2				
Set A	400			
Set B	400			
Set C	400			
Set D	400			
Set E	400			

### 3.1 Dataset – 1

Dataset – 1 consists of a synthetic, simulated action potential with additive Gaussian noise (Bernert and Yvert, 2019). The action potentials were generated based on Equation (9), and the details can be found elsewhere (Adamos et al., 2008).


(9) $$V\left(t\right)=Acos\left(2\pi \frac{t-\tau_{ph}}{\tau_{1}}\right)\exp\left(-{\left(\frac{2.3548t}{\tau_{2}}\right)}^{2}\right)$$

The selection of parameters for generating action potentials ( $A,\tau_{1},\tau_{2},\tau_{ph}$ ) are explained in Bernert and Yvert (2019). The dataset consists of seven different Signal-to-Noise Ratios (SNRs) – 0.5, 0.75, 1.0, 1.25, 1.50, 1.75, and 2.0. Each SNR value represents the level of Gaussian noise added to the signal. Each set contains three action potentials with different shapes and properties which are: N-class_1, N-class_2, N-class_3, and signal noise labeled N-class_4. For the experiment, only three Signal-to-Noise Ratio (SNR) values were chosen for testing: 0.5, 1.25, and 2.0. The test signal was generated with a sampling frequency of 20 kHz with a mean firing rate of 3.3 Hz. Each SNR set included ten recordings of 200 s. Table 2 shows the number of epochs for each class per SNR set. The size of each epoch is set to 56 samples. The preprocessed segmented datasets were used (Patel and Yildirim, 2023).

### 3.2 Dataset – 2

Dataset – 2 is a publicly available epilepsy EEG dataset that was recorded by the Department of Epilepsy at Bonn University, Germany (Andrzejak et al., 2001). It contains five different sets of recordings, labeled as E – Set_A, E – Set_B, E – Set_C, E – Set_D, and E – Set_E. Each set consists of 100 channels of EEG that were sampled at 173.61 Hz. Each set was recorded for 23.6 s for a total of 4,096 data points. Each channel was segmented into 4 epochs with 1,024 samples per epoch shown in Table 2. The EEG signal was bandpass filtered from 0.53 Hz to 85 Hz. Each EEG set was treated as an individual classifying class. Set A was scalp EEG recordings from healthy participants with eyes open, Set B was scalp EEG from healthy participants with eyes closed, Set C was interictal (between seizure) intracranial EEG recordings from hippocampal formations contralateral to the epileptogenic zone in mesial temporal epilepsy patients, Set D was interictal intracranial EEG recordings within the epileptogenic zone in mesial temporal epilepsy patients, and Set E was a recording of ictal (seizure) intracranial EEG activity from mesial temporal epilepsy patients.

## 4 Results and discussion

In this paper, GCFE method is compared with seven other feature extraction techniques. As shown in Table 1, all feature classification experiments conducted by this approach utilized a 1D-CNN model. The batch size was set to 32 for 1D-CNN model. All tests were conducted on a university supercomputer that was configured with 24 cores and 24 GB of memory per core. The consistent experimental setup ensures a valid assessment of the GCFE approach relative to other methods. The performance and accuracy scoring metrics for each experiment were determined using cross-validation.

Dataset – 1 was split into three groups: training, validation, and testing, with proportions of 70, 15, and 15%, respectively. The Dataset – 2 was split only into two groups: training (70%) and testing (30%). In Dataset – 2, the validation and testing datasets are kept the same size because of the limited number of epochs (instances). The decision to use these particular split ratios was guided by methodologies commonly adopted in other research papers, which served as benchmarks for comparison. Finally, each experiment was trained with 30 iterations.

**Figure 3:**
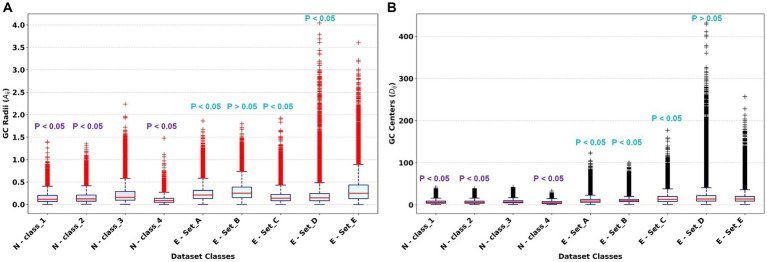
Box plot demonstrating the Distribution of GCFE using WVG for each dataset class, with statistical significance determined by the Wilcoxon rank sum test **(A)** Distribution of GC radii – Sum of weighted edges of $A_{ij}$ (row wise) vs. Dataset Classes; **(B)** Distribution of GC center – $D_{ij}$ vs. Dataset Classes.

Figures 3A,B show box plot distributions of reduced features for distinct dataset classes, specifically focusing on GC radii and GC center values, respectively. These figures also provide results from the Wilcoxon rank sum test under the null hypothesis that N-Class_4 from Dataset-1 and E-Set_E from Dataset-2 are superior to the remaining classes within their respective dataset groups. The distribution values were generated using the WVG method, incorporating 200 epochs randomly chosen from each class in Dataset-1(SNR – 0.5, 1.5, and 2.0) and from Dataset-2.

From Figure 3A, it can be shown that the GC radii values differ among classes. Specifically, N-class_3, characterized by a higher action potential amplitude, has significantly (*p* < 0.05) higher radii values compared to the action potentials of N-class_1 and N-class_2. Additionally, the Interquartile Range (IQR) of N-class_4 is considerably smaller than that of the other classes. This observation can be attributed to the fact that N-class_4 represents noise, which has a lower amplitude compared to other action potential classes. Consequently, the edges of the graph corresponding to N-class_4 are smaller, resulting in a smaller sum of weighted edges ( $A_{ij}$ ). The median $A_{ij}$ values for N-class_1, N-class_2, N-class_3, and N_class_4 were 0.12, 0.12, 0.16, and 0.08, respectively. A parallel pattern is noted in the GC centers for Dataset-1 classes as shown in Figure 3B, where N-Class_3 possesses the highest median $D_{ij}$ value of 7, significantly distinct from the other classes (*p* < 0.05). This is because N-Class_3 represents the action potential with the highest amplitude. According to the visibility graph concept, a graph representing this class will have more connections of edges with both near and far samples (nodes) of the signal, resulting into higher value of degree $(D_{ij}$ ) that is, greater GC centers values. The median values for GC centers for N-Class_1, N-Class_2, and N-Class_4 is 6, 6, and 5, respectively.

In Dataset-2, presented in Figure 3A, E-Set_B was characterized by increased radii values (*p* > 0.05) with a median $A_{ij}$ value of 0.25, comparable to E-Set-E which also had a median value of 0.25. The median GC radii values for E-Set_A, E-Set_C, and E-Set_D were 0.21, 0.14, and 0.15, respectively. Meanwhile, Figure 3B illustrates that, for Dataset-2, the GC Centers for E-Set_D were significantly different (*p* > 0.05) with a median value of 14. This pattern indicates that for non-stationary recordings with higher amplitude, both the GC radii and GC centers tend to exhibit higher median values. Conversely, for stationary or nearly stationary recordings with higher amplitude, the GC radii still display higher median values, but the GC centers tend to have lower median values. This observation is based on the standard weighted visibility graph theory, which helps differentiate the dynamic characteristics of the recordings based on their structural connectivity within the graph.

Table 3 presents eight different feature extraction studies represented as F1 (Mohammadpoory et al., 2023), F2 (Javaid et al., 2022), F3 (Supriya et al., 2016), F4 (Hao et al., 2016), F5 (Bose et al., 2020), F6 (Cai et al., 2022), F7 (Ahmadlou et al., 2010), and F8 (Proposed). In addition, it also illustrates the number of features extracted by each method per dataset. The features for each method were arranged in vector form. The F2 method has maximum features with 172 and 3,076 for Dataset – 1 and Dataset – 2, respectively. The GCFE (F8) for Dataset – 1 and Dataset – 2 were 112 and 2048 features, respectively. The F7 method feature count for Dataset – 1 was selected similarly to the F8 method, while for Dataset – 2, a maximum of 800 features were selected.

**Table 3:** Number of studies and its features counts per dataset.

Study name	Features	No. of features for Dataset – 1	No. of features for Dataset – 2
F1	GIC, CPL, GE, LE, CC, AC	61	1,029
F2	CC, CPL, AC, WD, GE, LE, NBC	172	3,076
F3	Modularity, CC	57	1,025
F4	CPL, CC	57	1,025
F5	CC, GE, LE, Transitivity	59	1,027
F6	WD, CC	112	2048
F7	PCA	112	800
F8 (Proposed)	GCFE	112	2048

Table 4 provides insights into each feature extraction method’s accuracy, sensitivity, and specificity across two distinct visibility graphs. Seven classification experiments were conducted using feature extraction methods F1 to F8. The F8 (proposed) method demonstrated superior performance over most of the other feature extraction methods, labeled F1 through F7. According to Table 4, it is evident that the F8 method outperformed the F2 method, which had the highest number of features. This outcome substantiates the assertion that an increase in the number of features does not necessarily enhance classification performance. Additionally, the F8 method achieved higher performance with fewer features, further illustrating the effectiveness of optimized feature extraction over mere quantity. Furthermore, the average accuracy differences computed to accurately compare the proposed feature extraction method’s performance against others.

**Table 4:** Summary of performance metrics for feature extraction studies using weighted visibility graphs and weighted dual perspective visibility graphs. the metrics are arranged in rows, in the order of accuracy, sensitivity, and specificity.

Experiments	F1	F2	F3	F4	F5	F6	F7	F8
*Weighted visibility graph*								
SNR 0.5	98.620	99.661	98.178	98.412	98.594	99.687	99.810	99.578
	98.620	99.661	98.178	98.412	98.594	99.687	99.810	99.578
	98.623	99.662	98.213	98.412	98.604	99.688	99.810	99.579
SNR 1.25	92.598	98.227	91.399	91.920	90.930	97.367	97.888	97.888
	92.598	98.227	91.399	91.920	90.930	97.367	97.888	97.888
	92.695	98.234	91.408	91.947	91.066	97.460	97.888	97.888
SNR 2.0	79.639	89.429	79.343	80.473	79.612	88.784	87.808	89.454
	79.639	89.429	79.343	80.473	79.612	88.784	87.808	89.454
	79.766	89.397	79.349	80.351	79.706	88.770	87.886	89.539
Set A vs. E	96.500	96.500	95.833	97.500	95.000	97.083	97.500	98.333
	96.500	96.500	95.833	97.500	95.000	97.083	97.500	98.333
	96.531	96.342	96.057	97.559	95.095	97.118	97.559	98.391
Set B vs. E	94.166	94.583	96.666	96.666	96.666	97.916	95.416	97.916
	94.166	94.583	96.666	96.666	96.666	97.916	95.416	97.916
	94.392	95.151	96.726	96.795	96.683	97.950	95.453	98.006
Set C vs. E	95.000	97.916	95.000	96.250	93.333	98.750	96.666	97.916
	95.000	97.916	95.000	96.250	93.333	98.750	96.666	97.916
	95.131	97.950	95.037	96.342	93.417	98.754	96.710	97.950
Set D vs. E	94.166	96.250	94.583	91.666	95.833	96.666	96.250	98.333
	94.166	96.250	94.583	91.666	95.833	96.666	96.250	98.333
	94.186	96.286	94.995	91.692	95.894	96.726	96.286	98.333
*Weighed dual perspective visibility graph*								
SNR 0.5	98.516	99.609	98.282	98.230	98.074	99.661	99.831	99.493
	98.516	99.609	98.282	98.230	98.074	99.661	99.831	99.493
	98.532	99.611	98.291	98.238	98.087	99.662	99.831	99.498
SNR 1.25	91.920	98.123	92.025	91.842	91.712	97.993	97.973	98.163
	91.920	98.123	92.025	91.842	91.712	97.993	97.973	98.163
	91.903	98.132	92.007	91.947	91.923	98.000	97.973	98.163
SNR 2.0	81.011	89.133	79.639	79.747	79.935	89.187	87.873	89.346
	81.011	89.133	79.639	79.747	79.935	89.187	87.873	89.346
	81.120	89.394	79.531	79.730	79.982	89.228	87.789	89.442
Set A vs. E	97.500	96.250	96.666	97.500	96.666	95.833	97.916	98.333
	97.500	96.250	96.666	97.500	96.666	95.833	97.916	98.333
	97.559	96.255	96.666	97.559	96.890	96.057	98.006	98.391
Set B vs. E	93.333	97.083	91.666	89.583	97.500	92.916	92.083	97.083
	93.333	97.083	91.666	89.583	97.500	92.916	92.083	97.083
	93.675	97.256	92.938	91.498	97.516	93.857	92.391	97.174
Set C vs. E	95.833	97.916	95.416	95.833	95.000	97.916	97.083	97.916
	95.833	97.916	95.416	95.833	95.000	97.916	97.083	97.916
	95.851	97.921	95.416	95.833	95.131	97.950	97.088	98.006
Set D vs. E	95.833	96.666	95.833	94.583	89.166	97.500	94.166	97.083
	95.833	96.666	95.833	94.583	89.166	97.500	94.166	97.083
	96.057	96.795	96.057	94.621	89.486	97.559	94.230	97.118

**Figure 4:**
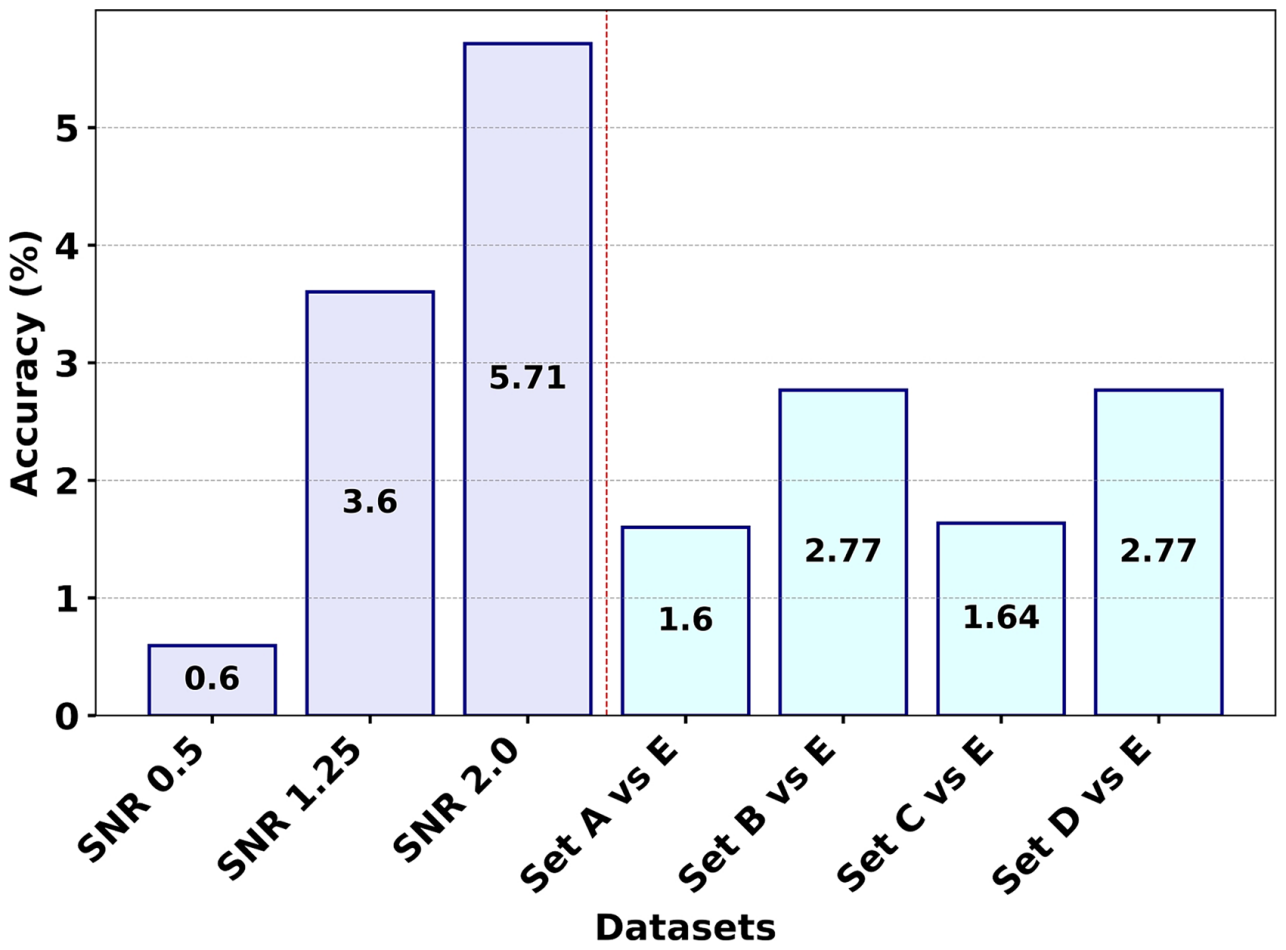
Comparative analysis of the average accuracy differences for the F8 (GCFE) method using WVG and WDPVG graph types across seven experiments.

Figure 4 presents a visual comparison of the average accuracy differences for the F8 method across seven studies, utilizing both WVG and WDPVG techniques. The process involved calculating the mean accuracies for WVG and WDPVG in each experiment, followed by determining the average difference in accuracy between the F8 method and the other studies. Figure 4 shows that experiment F8 consistently outperforms the F1 to F7 methods by having a positive average accuracy difference across all datasets. Among all the experiments, the F8’s performance for Set A vs. Set E had the lowest average accuracy difference. Additionally, in the SNR dataset experiment, F8 shows robustness and superior performance, especially as the signal became noisier (at SNR 2.0) compared to other methodologies.

**Figure 5:**
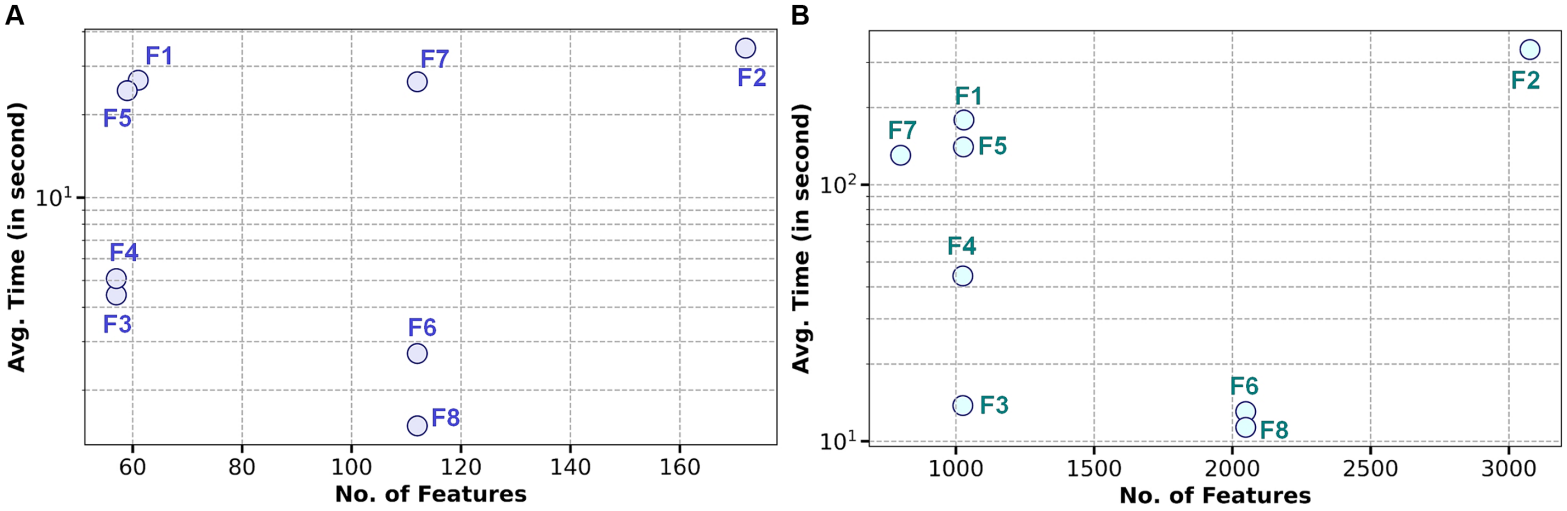
Representation of average (avg.) computational time for both datasets vs. number of features in each feature extraction method: **(A)** Log-scale avg. computational time vs. number of features in Dataset – 1; **(B)** Log-scale avg. computational time vs. number of features in Dataset – 2.

Figure 5 presents the average computational time for each feature extraction method across the two datasets. Figure 5A represents Dataset – 1, and Figure 5B represents Dataset – 2. In both representations, the *x*-axis denotes the number of features for each method. The *y*-axis displays the average computation time for each method in seconds on a logarithmic scale. The computation time is calculated for each method for an average of 25,325 and 800 epochs for Dataset – 1 and Dataset – 2, respectively.

Similar trends were observed in Figure 5B for Dataset 2, where the F8 method was the most time-efficient, averaging 11.34 s. In contrast, the F2 method was the most time-consuming, requiring an average of 336.69 s. Despite having fewer features, as indicated in Table 3, the methods F1, F3, F4, F5, and F7 still demanded more time than F8, with average times of 178.94 s, 13.79 s, 44.11 s, 140.46 s, and 130.39 s, respectively. The F6 method was the only feature extraction method that came close to F8 in terms of computation time, averaging 13.06 s. From Tables 4 and 5, it is revealed that possessing a larger number of extracted features, specifically the F2 method with the most features, does not enhance classification accuracy and leads to increased computational time. All the results and supporting code are made available on GitHub (Patel, 2023).

**Table 5:** Computational times (in seconds) for feature extraction studies using weighted visibility graphs and weighted dual perspective visibility graphs across various experiments.

Experiments	F1	F2	F3	F4	F5	F6	F7	F8
*Weighted visibility graph*								
SNR 0.5	27.4	33.7	4.3	4.5	25.3	2.6	27.4	1.5
SNR 1.25	22.4	30.5	4.1	4.2	19.9	2.6	26.5	1.5
SNR 2.0	19.6	26.6	4.0	4.1	17.6	2.5	25.1	1.5
Set A vs. E	104.7	246.2	13.2	37.8	76.7	12.8	128.3	10.8
Set B vs. E	105.7	247.2	13.7	37.7	76.4	13.6	133.0	11.3
Set C vs. E	124.6	285.2	13.9	40.6	94.9	12.3	129.5	11.2
Set D vs. E	176.9	345.3	13.8	42.2	141.9	12.2	131.3	11.5
*Weighed dual perspective visibility graph*								
SNR 0.5	35.2	45.5	5.1	6.1	34.3	3.2	27.6	1.5
SNR 1.25	28.9	38.2	5.0	6.2	25.8	2.8	26.6	1.5
SNR 2.0	26.6	35.0	4.1	5.4	23.9	2.6	24.9	1.4
Set A vs. E	159.4	314.9	14.5	44.8	120.9	12.9	131.8	11.5
Set B vs. E	164.3	306.7	13.1	49.5	119.3	13.7	131.6	11.2
Set C vs. E	208.2	346.9	14.5	49.0	142.0	13.9	127.2	11.4
Set D vs. E	387.7	601.1	13.6	51.3	351.6	13.1	130.4	11.8

## 5 Conclusion

In conclusion, this paper demonstrated a new implementation of the GC theorem with the mWL matrix as a feature extraction methodology for biomedical signals. In addition, the results clearly support that the GCFE approach surpasses other feature reduction techniques. Additionally, GCFE delivered consistently positive average accuracy difference across both datasets and two distinct graphs. Further, the computational efficiency of the proposed methodology was better when compared to other methods. The superior accuracy and decreased computational time of GCFE demonstrates that it exceptionally well-suited for real-time biomedical signal classification applications. However, the proposed GCFE is constrained to extracting a fixed number of features, converting an N x N Laplacian matrix to a 2 × N vector due to its non-parametric approach. Future research could expand the potential uses of GCFE by integrating alternative eigenvalue inclusion theorems or by modifying the GC theorem to predict more precise eigenvalue inclusions of random Laplacian matrices.

## Data availability statement

The original contributions presented in the study are included in the article/Supplementary material, further inquiries can be directed to the corresponding author.

## Author contributions

SP: Conceptualization, Formal analysis, Methodology, Validation, Writing – original draft, Writing – review & editing. RS: Formal analysis, Funding acquisition, Investigation, Project administration, Resources, Supervision, Validation, Visualization, Writing – review & editing. AY: Formal analysis, Investigation, Project administration, Resources, Supervision, Validation, Visualization, Writing – review & editing.
