# Supplementary material for: Gershgorin circle theorem-based feature extraction for biomedical signal analysis
Source: Front Neuroinform. 2024 May 16;18:1395916. doi: 10.3389/fninf.2024.1395916 (PMC11137292; doi:10.3389/fninf.2024.1395916)
Supplement: Supplementary file 1 [file Data_Sheet_1.PDF]

Supplementary Material

Gershgorin Circle Theorem-Based Feature Extraction for Biomedical Signal Analysis

Sahaj A. Patel[1*], Rachel June Smith[1], Abidin Yildirim[1]

*** Correspondence:** Sahaj A. Patel; sahaj432@uab.edu

1 Dataset-1 Class Visualization

Supplementary Figure 1 provides a visual representation of each class in Dataset 1 at an SNR of 0.5. N-Class_1 and N-Class_2 exhibit similar amplitudes in their action potentials or neural spikes but differ in their phase differences. In contrast, N-Class_3 displays a higher amplitude compared to N-Class_1 and N-Class_2. Meanwhile, N-Class_4 represents random signal noise, with the same sample size as the other classes in the dataset.

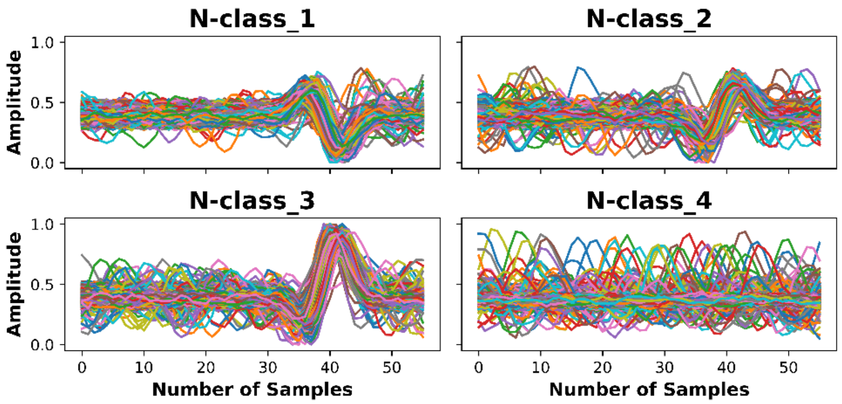

Supplementary Figure 1: Comparative visualization of action potential variability across four neural classes at an SNR of 0.5.

2 Gershgorin Circle Theorem and Importance of Strictly diagonal Matrix

The example below illustrates the importance of the modified Laplacian matrix, which possesses the property of being strictly diagonally dominant, as proposed in this study, and how it impacts the Gershgorin Circle features.

Consider the matrix X which is neither diagonally strictly dominant nor symmetric:

$$X = \begin{bmatrix} 0.52 & 0.57 & 0.71 \\ 0.53 & 0.13 & 0.15 \\ 0.36 & 0.67 & 0.82 \end{bmatrix}$$

The eigenvalues of X, $\sigma(X)$, are computed as:

$$\sigma(X) = \{1.510 + 0.j, -0.0201 + 0.0546j, -0.0201 - 0.0546j\}$$

Let's apply the Gershgorin Circle (GC) Theorem to matrix X. The centers $C(X)$ and the radii $R(X)$ of the Gershgorin circles for matrix X are defined as follows:

$$R(X) = \{r_1(X), r_2(X), r_3(X)\}$$
$$= \{(0.57 + 0.71), (0.53 + 0.15) + (0.67 + 0.82)\}$$
$$= \{1.28, 0.68, 1.49\}$$
$$C(X) = \{c_1(X), c_2(X), c_3(X)\}$$
$$= \{0.52, 0.13, 0.82\}$$

As illustrated in Supplementary Figure 2, all eigenvalues of matrix X are located on the real and imaginary axes, and the circles with dots inside Supplementary Figure 2 represent the approximate eigenvalue inclusion for matrix X. According to the Gershgorin Circle (GC) Theorem, the union of all Gershgorin Circles represents the eigenvalue inclusion zones. In the case of matrix X, if the matrix were symmetric (as in the case of a Laplacian matrix), then all eigenvalues would be located on the real axis. However, symmetry alone does not guarantee whether the matrix will be singular or non-singular.

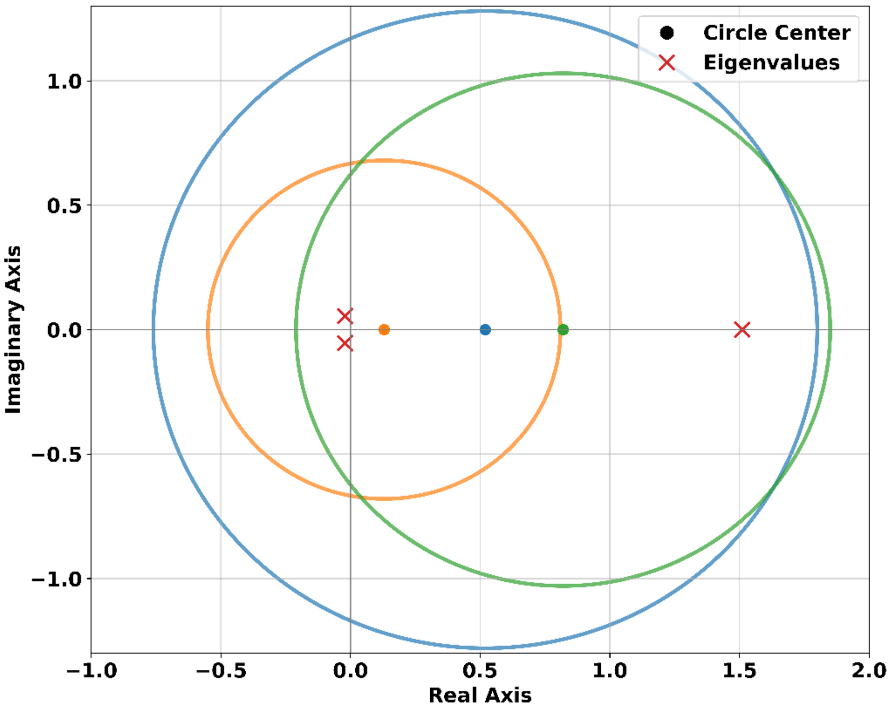

Supplementary Figure 2: Visualization of Gershgorin Circles and Eigenvalues for Non-strictly Diagonal Matrix.

To address potential issues of singularity, this study proposes the use of a modified Laplacian matrix that is strictly diagonally dominant. This structural modification ensures that the matrix is always non-singular, meaning it will always have non-zero eigenvalues, based on the Gershgorin Theorem.

Supplementary Figure 3 demonstrates the eigenvalue inclusion for matrix Y, a symmetric and strictly diagonally dominant matrix where all eigenvalues are non-zero and lie on the real axis.

$$Y = \begin{bmatrix} 0.58 & 0.12 & 0.40 \\ 0.02 & 0.10 & 0.45 \\ 0.40 & 0.45 & 0.89 \end{bmatrix}$$

The eigenvalues of Y, $\sigma(Y)$, are computed as:

$$\sigma(Y) = \{1.1033, 0.2604, 0.0962\}$$

The center $C(Y)$ and radii $R(Y)$, of the GCs for matrix Y are defined as follow:

$$R(Y) = \{r_1(Y), r_2(Y), r_3(Y)\}$$

$$= \{(0.12 + 0.40), (0.12 + 0.45) + (0.40 + 0.45)\}$$

$$= \{0.52, 0.57, 0.85\}$$

$$C(Y) = \{c_1(Y), c_2(Y), c_3(Y)\}$$

$$= \{0.58, 0.6, 0.89\}$$

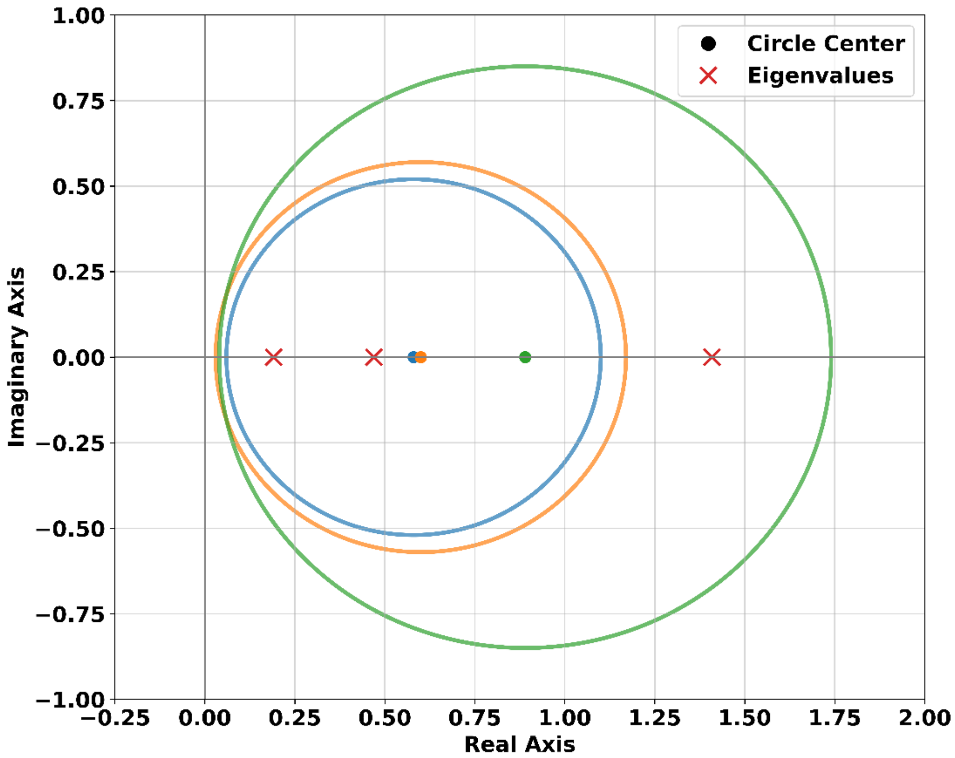

Supplementary Figure 3: Visualization of Gershgorin Circles and Eigenvalues for Strictly Diagonal Matrix.
